# The Temptation of Economical Management

**Published:** 1918-02-23

**Authors:** 


### THE RECORD OF THE RED CROSS.

### The Temptation of Economical Management.

In our review of the previous Report of the British Red Cross Society and the Order of St. John, of which the third yearly statement has now been published, we drew attention to the balance in hand with which the year 1917 began. The development of the war on the various Fronts led the expenditure to exceed the estimate by a large figure, but it is good to know that the Joint Committee again possesses a balance. The fact is worth noting for two reasons. First, the income derived from the investment of the surplus was practically sufficient to pay the entire cost of administration, which thus did not encroach directly on the funds subscribed for the wounded. Again, though a balance exists, last year was the first to show a deficit. A serious falling off has occurred in the normal daily subscriptions. This means that to meet the still growing expenditure special efforts again will have to be made to secure the position, and to provide a fund to maintain hospitals and services after peace has been declared, when the influx of subscriptions is expected to stop immediately.

With this glance at the general position, let us turn to some of the details of the work disclosed in the Report, for these bring home to us the ramifications of a work whose total magnitude dulls rather than excites the imagination when expressed in mere millions.

The most expensive and costly department of the whole organisation is now that of stores, which supplies the wounded with comforts of every description. On one occasion, when a surgical instrument was wanted for a special operation during an interruption of the Channel service, the Stores Department, through the Transport Office at Folkestone, delivered the instrument by aeroplane within a few hours of the arrival of the telegram. Large sums have been voted to establish orthopædic centres, and the Society by helping to provide the equipment on the understanding that the War Office would maintain the centres when organised, has helped to establish these without unnecessary delay. The War Library continues to grow with the increasing demand for books and papers, and it is curious to learn that the difficulty in binding and the shortage of supplies have prevented or hindered purchases. The department known as the Central Prisoners of War Fund, the aim of which is to secure to every British prisoner of war the supply of food prescribed by the War Office, supervises packing for over 15,000 prisoners and more than 600 officers. The total number of prisoners is given as 55,000, and stores on a large scale have to be purchased in advance for them. Like another Cook, the department in charge of relatives' visits to prisoners transported thirty-seven parties, averaging sixteen in number, to Switzerland and billeted them there for about a fortnight.

A little known branch of the Joint Committee's work is the Compassionate Fund for Red Cross *personnel*. During the year under review 102 applications for assistance were received, and 92 grants were made at a cost of £1,222. Both the number of applications and the amount expended on the grants seems to us surprisingly small. We hope that applications are considered sympathetically, and that the grants, to say the least, are worthy of the professed motive of a Red Cross Fund. The point is worth making, for one of the bad old legacies from nineteenth-century commercialism still lingers in our institutions—namely, that the chief source of pride in charitable undertakings should be the small cost of administration. One had thus institutions founded to relieve some class of sufferer which by under-payment of their own staffs, and neglect to provide for them in their old age or misfortune, created another class of sufferers under the infamous plea of economical management. The Red Cross Society, we hope, is setting up quite a different precedent, for we cannot but suppose there must be many Red Cross workers whom illness or misfortune should rightly throw upon the generosity of this Compassionate Fund. Only the other day a well-meaning philanthropist left a large sum of money to provide pensions of £20 a year to necessitous ladies. Of what use, save to exacerbate poverty, is such a pension? Nothing less than a pound a week is the smallest real relief to anyone, and the philanthropist's narrowness of view is typical of a still surviving and niggardly fashion. Against this we hope that the Joint Societies' Compassionate Fund will be a standing protest, and consequently a source of blessing not only to its beneficiaries, but to countless others influenced by the standard adopted by the Red Cross.

In conclusion we may note that the work of the Graves Restoration Fund proceeds steadily, and that by a weekly grant of £100 the graves of those buried in France are cared for and photographed, while arrangements are being made to establish a department in France to draft ground plans, and provisionally to secure the permanent care of the cemeteries. From the first general statement to this detail the Report is an inspiring record of work, the application to which of the hospital standard requires that the workers in the vineyard of the fund shall be as generously treated as the objects of it. This warning is not ungracious while one remembers how grossly underpaid trained nurses have been, and still are, in voluntary hospitals.

For Editor's Letter Box, see p. 447.

